# The Emerging Role of OTUB2 in Diseases: From Cell Signaling Pathway to Physiological Function

**DOI:** 10.3389/fcell.2022.820781

**Published:** 2022-03-02

**Authors:** Jun Li, Na Zhang, Meihua Li, Tao Hong, Wei Meng, Taohui Ouyang

**Affiliations:** ^1^ Department of Neurosurgery, The First Affiliated Hospital of Nanchang University, Jiangxi, China; ^2^ Department of the Second Clinical Medical College of Nanchang University, Jiangxi, China; ^3^ Department of Neurology, The First Affiliated Hospital of Nanchang University, Jiangxi, China

**Keywords:** OTUB2, ubiquitination, cell signaling pathways, cancer, physiological function

## Abstract

Ovarian tumor (OTU) domain-containing ubiquitin aldehyde-binding protein Otubain2 (OTUB2) was a functional cysteine protease in the OTU family with deubiquitinase activity. In recent years, with the wide application of molecular biology techniques, molecular mechanism regulation at multiple levels of cell signaling pathways has been gradually known, such as ubiquitin-mediated protein degradation and phosphorylation-mediated protein activation. OTUB2 is involved in the deubiquitination of many key proteins in different cell signaling pathways, and the effect of OTUB2 on human health or disease is not clear. OTUB2 is likely to cause cancer and other malignant diseases while maintaining normal human development and physiological function. Therefore, it is of great value to comprehensively understand the regulatory mechanism of OTUB2 and regard it as a target for the treatment of diseases. This review makes a general description and appropriate analysis of OTUB2’s regulation in different cell signaling pathways, and connects OTUB2 with cancer from the research hotspot perspective of DNA damage repair and immunity, laying the theoretical foundation for future research.

## 1 Introduction

Ubiquitin is a small molecule protein widely present in eukaryotic cells and plays an essential role in cells by modifying related proteins (Swatek and Komander, 2016). Ubiquitination is a significant protein post-translational modification, which affects the degradation and localization of associated proteins, the regulation of cell signaling pathways, and the selection of DNA damage repair pathways (Uckelmann and Sixma, 2017; Mansour, 2018; Park et al., 2020). The ubiquitination process is that ubiquitin is covalently bound to the target protein in the form of single-site monoubiquitination, monoubiquitination at multiple sites, and polyubiquitin chain under the ordered cascade of enzymatic reactions of E1 ubiquitin-activating enzymes, E2 ubiquitin-conjugating enzymes, and E3 ubiquitin ligases (Wang et al., 2017). Generally, E2 ubiquitin-conjugating enzymes determine the binding type of ubiquitination, and E3 ubiquitin ligases have a high degree of specificity in selecting substrate proteins (Metzger et al., 2014; Zheng and Shabek, 2017; Ullah et al., 2019).

Ubiquitination is vital *in vivo* due to its diverse functions in cells (Zheng and Shabek, 2017). However, it is antagonized by the deubiquitination mediated by deubiquitinating enzymes (DUBs) which can reverse the effect of E3 ligases and detach ubiquitin from the target protein (Satija et al., 2013). The dynamic balance between ubiquitination and deubiquitination in cells maintains normal physiological functions of the human body, and their imbalance can lead to many diseases, such as cancers (Satija et al., 2013). Currently, the known DUBs are mainly divided into seven categories including the ovarian tumor proteases (OTUs) superfamily (Hermanns and Hofmann, 2019). According to the molecular structure of proteins, the OTUs superfamily could be subdivided into four subfamilies: OTU deubiquitinating with linear linkage specificity (OTULIN), A20-like subfamily, ovarian tumor domain-containing protein subfamily, ubiquitin aldehyde binding protein subfamily with OTU domain (Otubains) (Keusekotten et al., 2013).

Otubain2 (OTUB2) is a representative member of the Otubains subfamily. Accumulating evidence has shown that OTUB2 plays a non-negligible role *in vivo* through deubiquitinating other proteins. In this review, we start with the molecular structure and function of OTUB2, compare the similarities and differences between OTUB2 and other members of the Otubains subfamily, and summarize the role and molecular mechanism of OTUB2 in physiological and pathological processes. Furthermore, we have rationally conceived the treatment plan targeting OTUB2, and proposed research directions for OTUB2.

## 2 The Molecular Structure and Function of OTUB2

### 2.1 The Similarities Between OTUB2 and Otubains Subfamily

OTUB2 is a protein molecule whose molecular structure consists of a five-stranded *β*-sheet in the middle, and a small helical amino-terminal region (α1, α2) and a large helical region (α3-α8) on both sides (Nanao et al., 2004). The active site of OTUB2 is at the junction of the *β*-sheet and *α*-helix and contains the catalytically active Cys51 residues and His224 residues shared with other Otubains family members such as OTUB1 (Balakirev et al., 2003), so Otubains are also called functional cysteine proteases. Although the Otubains are functionally similar to cysteine proteases, their spatial structure is not completely identical. For example, OTUB1 and OTUB2 are both based on the formation of oxyanion holes by the combination of the amide of cysteine and the amide of aspartic acid, forming a special spatial conformation different from the cysteine proteases family (Nanao et al., 2004; Edelmann et al., 2009).

### 2.2 The Differences Between OTUB2 and Otubains Subfamily

In terms of domain, OTUB2 only contains a carboxyl-terminal OTU catalytic active domain, but OTUB1 has one more amino-terminal ubiquitin-binding domain, which provides a structural basis for its interaction with E2 enzymes and can buffer or even inhibit ubiquitination events in cells (Nakada et al., 2010; Du et al., 2020). The lack of domain number in protein molecules may prevent OTUB2 from having various post-translational modifications such as hydroxylation and phosphorylation as other deubiquitination enzymes (Zhu Q. et al., 2021). Nowadays, for the modification regulation of OTUB2, only SUMOylation of the Lys233 site of OTUB2 is confirmed to interact with Yes-associated protein/transcriptional co-activator with PDZ-binding motif (YAP/TAZ), thereby enhancing the activity of OTUB2 and mediating the transfer of ubiquitin (Zhang et al., 2019). In terms of catalytic activity, although OTUB2 is highly similar to other DUBs in some catalytic residues, unlike Asp in OTUB1, Asn226 constitutes the third residue of the catalytic triad of OTUB2, which is significant for the correct alignment and polarization of histidine (Clague et al., 2013; Sivakumar et al., 2020). Asn226 orients the side chain of the catalytic histidine to the appropriate position of each step of the catalytic mechanism to play a key auxiliary role (Vernet et al., 1995), proved by the decrease of deubiquitinase activity when point mutation technology (Asn226 mutated to Ala) is used (Balakirev et al., 2003). Meanwhile, Asn226 can form a hydrogen bond network between Thr45 and His224 in OTUB2 (Nanao et al., 2004). Interestingly, there is a novel oxyanion hole inside OTUB2 generated by the active site loop, which may be conducive to the interaction between OTUB2 and ubiquitin (Nanao et al., 2004).

**FIGURE 1:**
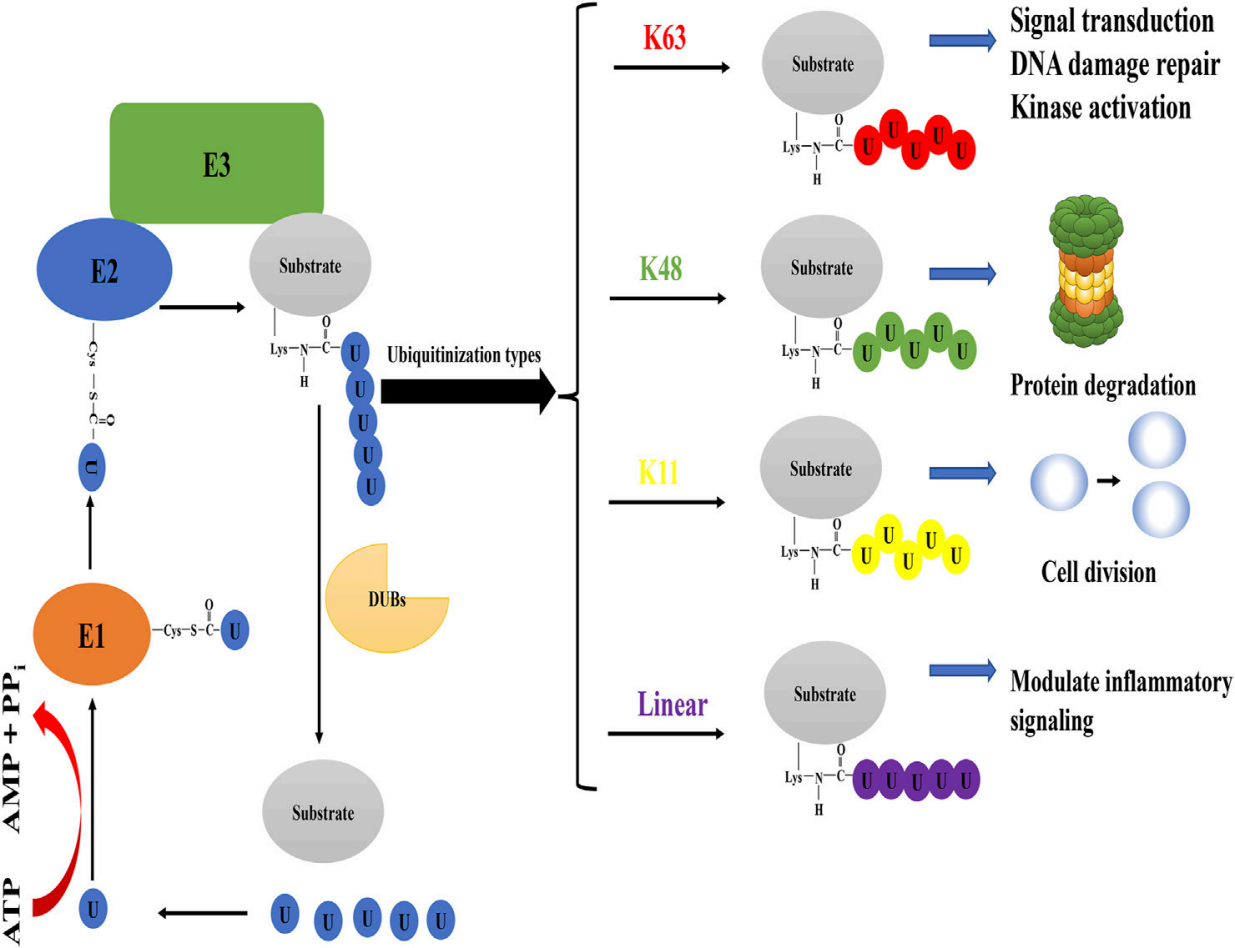
The process of protein ubiquitination and biological events mediated by some ubiquitination types. In the case of ATP supplying energy, E1 ubiquitin-activating enzyme activates ubiquitin molecules. E1 ubiquitin-activating enzyme transfers the activated ubiquitin molecules to E2 ubiquitin-conjugating enzyme, and the cysteine active site of E2 ubiquitin-conjugating enzyme forms thioester bonds with ubiquitin transferred by E1 ubiquitin-activating enzyme. E3 ubiquitin ligase participates in the ubiquitination of substrate by forming an isopeptide bond between the carboxyl group of Gly76 of ubiquitin and the ε-amine of Lys in the substrate. Typical types of polyubiquitination are K48 (ubiquitin is linked to ubiquitin at lysine 48 residue to form the isopeptide-linked polyubiquitin chain) and K63 (lysine 63 residue). The former mainly performs the degradation of proteins in cells, while the latter has achievements in DNA damage repair, kinase activation, signal transduction, and so on. Atypical ubiquitin chains are K11 (lysine 11 residue) and linear ubiquitin chain (the N-terminus of the ubiquitin molecule is directly connected to the C-terminus of another ubiquitin molecule to form a peptide bond). OTUB2 can also cleave the K11 polyubiquitin chain, which may have a role in the regulation of cell division. In addition, the linear ubiquitin chain often plays an activating role in cellular pathways that regulate inflammatory responses and can be specifically cleaved by OTULIN. Note: Different colors of ubiquitin molecules in the figure represent different types of ubiquitination.

It is well known that ubiquitin has seven lysine residues, which makes it possible for ubiquitins to connect through different lysine residues to form multiple polyubiquitin chains. More importantly, the N-terminal of ubiquitin can also be linked with the C-terminal of another ubiquitin to form peptide bonds, thus forming a new linear polyubiquitin chain (Kirisako et al., 2006). The polyubiquitin chain is linked to the lysine residue at the N-terminal of the substrate protein through the glycine at the C-terminal to complete the ubiquitination of the substrate. This complex reaction requires a variety of enzymes to cooperate (Figure 1). According to the different types of polyubiquitin chains, their biological functions are also different. The well-known Lys48-linked (K48) polyubiquitin chain usually guides the proteasome pathway to degrade substrate proteins (Pickart, 2004). Conversely, the Lys63-linked (K63) polyubiquitin chain mediates a wide range of biological functions, such as DNA damage repair, cell signaling, intracellular trafficking, and translation (Pickart and Eddins, 2004; Bennett and Harper, 2008; Chen and Sun, 2009). Current studies have shown that OTUB2 has cleavage activity on differently linked polyubiquitins such as the Lysine-48 (K48), the Lysine-63 (K63) and the Lysine-11 (K11) polyubiquitin chain (Mevissen et al., 2013; Kato et al., 2014; Li et al., 2019a), whereas OTUB1 is only effective on K48 polyubiquitin chains in terms of its catalytic activity (Mevissen et al., 2013). The reason for selective cleavage of ubiquitin chains may be partly due to the N-terminal nature of Otubains (Edelmann et al., 2009). The N-terminal *α*-helix of OTUB1 is in direct contact with the proximal ubiquitin, thus confining its binding to an orientation presenting Lys48 towards the catalytic site. In contrast, the N-terminal tail of OTUB2 is shorter than OTUB1 and lacks the 37 residues present at the N-terminal of OTUB1, thus possibly lacking this feature to control cleavage specificity (Altun et al., 2015).

## 3 The Relationship Between OTUB2 and Diseases

### 3.1 OTUB2 Causes Diseases Such as Cancer by Affecting Cell Signaling Pathways

#### 3.1.1 The Hippo Pathway

**FIGURE 2:**
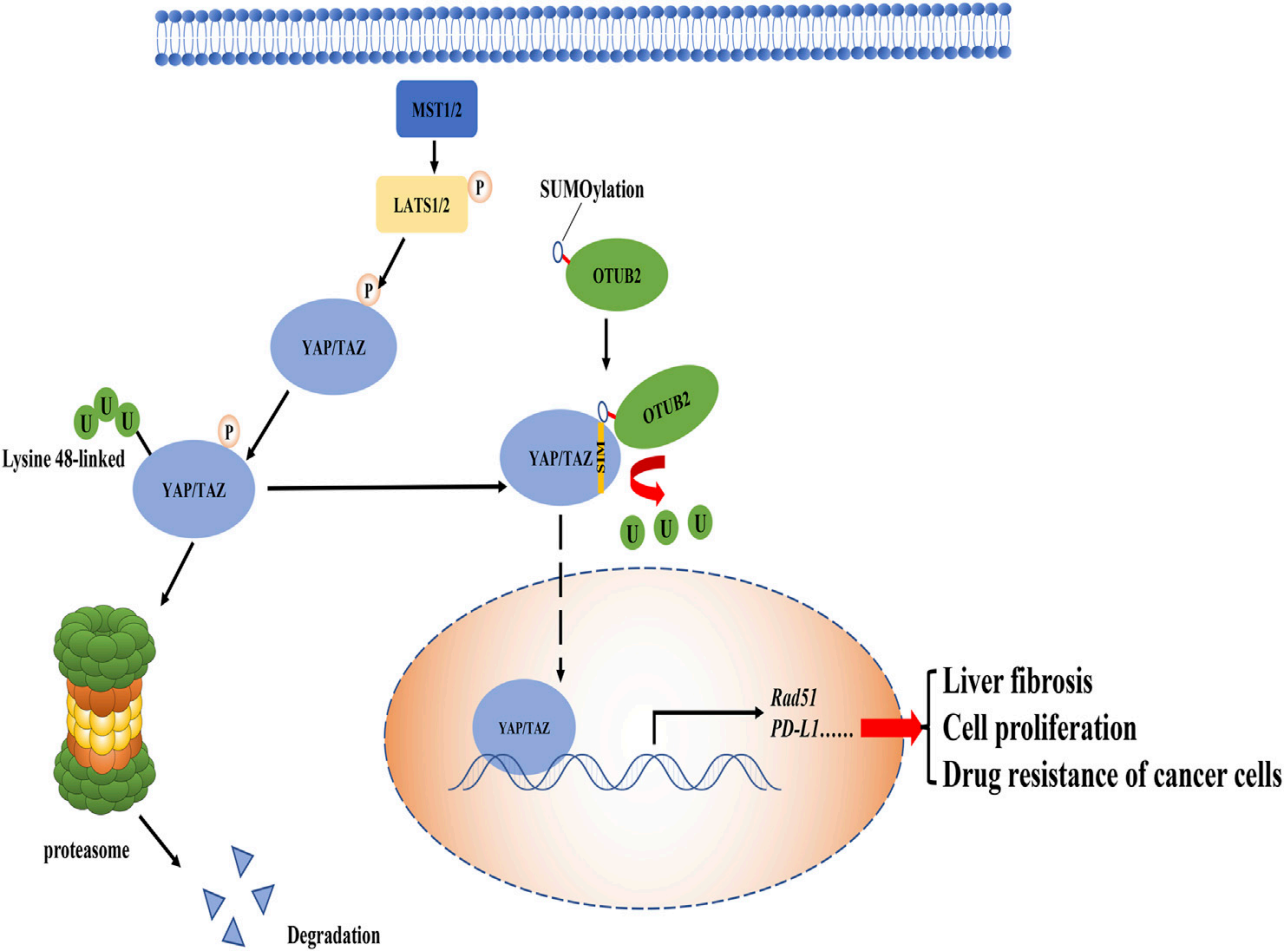
The role of OTUB2 in the Hippo pathway. OTUB2 was SUMOylated in the case of *RAS* gene mutation and interacted with the SIM of YAP/TAZ, thereby reducing the presence of the K48 polyubiquitin chain on YAP/TAZ. Undergraded YAP/TAZ entered the nucleus to stimulate the expression of downstream genes, causing a series of reactions that were not conducive to the normal physiological function of the human body.

The downstream molecule YAP/TAZ in the Hippo pathway is a transcription factor that can be transported from the cytoplasm to the nucleus and enhance the expression of various oncogenes, exerting its cancer-promoting effect. (Zhao et al., 2010). When the Hippo pathway is activated, YAP/TAZ is phosphorylated and stabilized in the cytoplasm and then degraded by the K48 polyubiquitin chain modification (Pan, 2010). The YAP/TAZ deubiquitination of OTUB2-mediated was discovered by Zhang et al.. In mouse breast cancer cells, OTUB2 is SUMOylated at the Lys233 site after translation, and modified OTUB2 relies on this modification to interact with SUMO-interacting motif (SIM) of YAP/TAZ to perform its deubiquitination function, ultimately affecting cancer metastasis (Figure 2). It is worth mentioning that the small ubiquitin-like modifier (SUMO) is probably caused by mutations in the *RAS* oncogene (Zhang et al., 2019). Therefore, OTUB2, as an intermediate molecule, both undertakes the downstream effects of *RAS* mutations and triggers YAP/TAZ-mediated oncogene expression based on its deubiquitination function (K48), forming a chain reaction and worsening cancer progression. On this basis, the overexpression of recombination protein A (Rad51) in endometrial cancer is a representative example of YAP/TAZ-mediated gene expression (Wan et al., 2020). The up-regulation of Rad51 protein can not only lead to drug resistance of cancer cells but also promote cell growth and proliferation (Klein, 2008; Chen et al., 2017). Not limited to cancer, the latest research has shown that pantoprazole promotes the degradation of YAP and finally blocks the progress of liver fibrosis by breaking the interaction between OTUB2 and YAP, which indirectly demonstrates that OTUB2 contributes to the progression of liver fibrosis (Lu et al., 2020).

In summary, the deubiquitination of YAP/TAZ by OTUB2 is fundamental to the downstream effects in the Hippo pathway. Notably, in the studies of OTUB2 targeting YAP/TAZ, there are various indications that this deubiquitination should be directed to the K48 polyubiquitin chain type. Although OTUB2 may promote DNA damage repair, and the selection of DNA damage repair pathway is often related to the K63 polyubiquitin chain of substrate protein, it cannot fully illustrate that OTUB2 directly targets the K63 type polyubiquitin chain. Because the biological effects of DNA damage repair may be controlled by OTUB2-mediated K48 deubiquitination downstream protein, in other words, OTUB2 rescues the degradation of proteins that induce DNA damage repair. Therefore, the selection and regulation of ubiquitination are often important for a comprehensive understanding of OTUB2.

#### 3.1.2 The NF-kappaB Pathway

**FIGURE 3:**
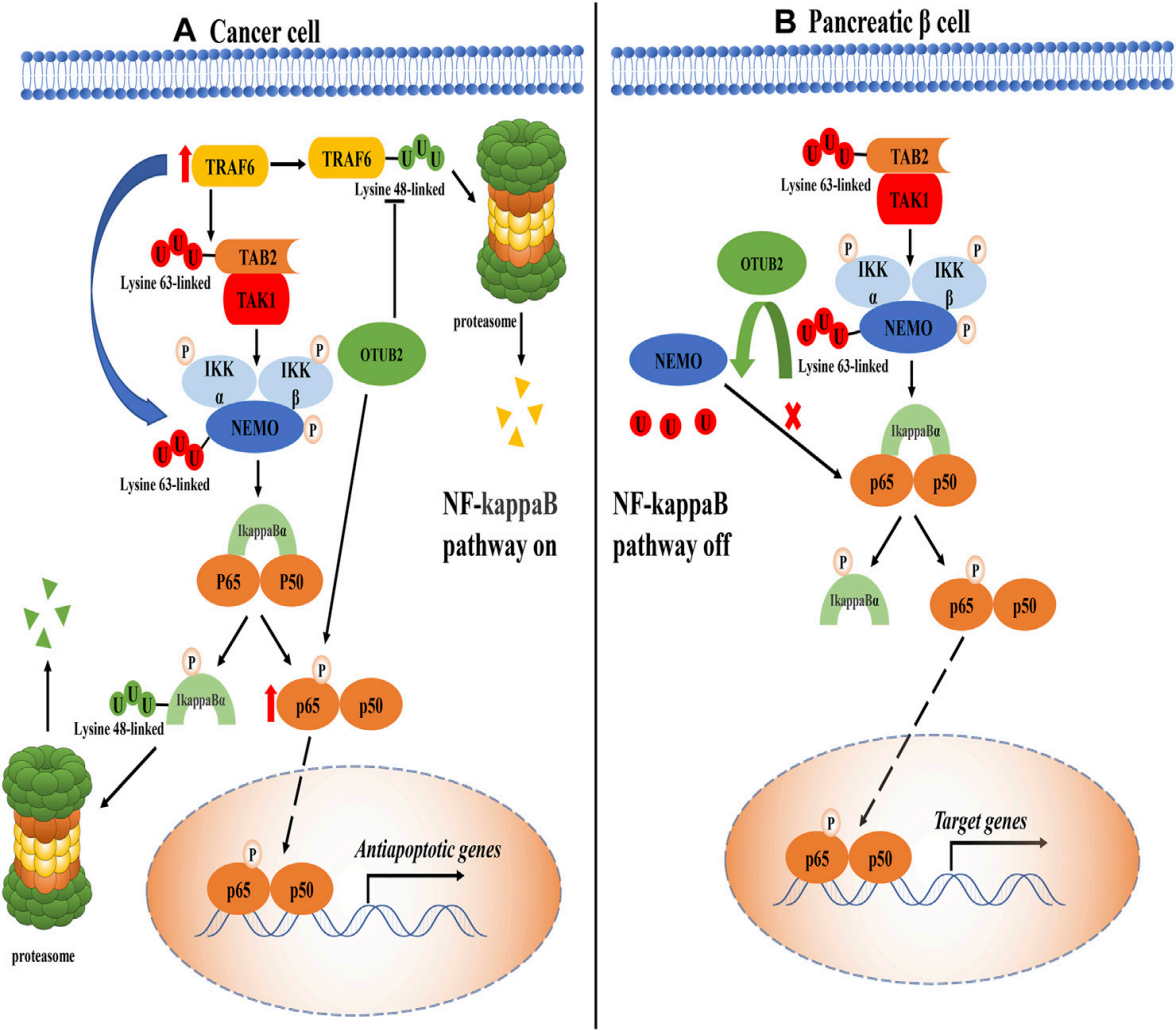
The role of OTUB2 in the NF-kappaB pathway. **(A)**. In cancer tissue, OTUB2 could not only remove the K48 polyubiquitin chain bound to TRAF6 but also increase the level of phosphorylated p65 (The intermediate pathway may be OTUB2-mediated K48 deubiquitination of phosphokinase targeting p65, not shown in the figure). As an E3 ubiquitin ligase, TRAF6 could ubiquitinate the key molecules TAB2 and NEMO in the NF-kappaB pathway. The K63 polyubiquitination chain of TAB2 interacted with the regulatory subunit of TAK1 to initiate the catalytic activity of TAK1. TAK1 made the phosphorylation activation of the IKK complex inevitable. The IKK complex dissociated the downstream molecule inhibitor of kappaB alpha (IkappaBα) from NF-kappaB (p-p65 + p50). The next fate of IkappaBα in isolation was added with the K48 polyubiquitin chain followed by proteasome degradation. Then the NF-kappaB escaped the restriction of the IkappaBα, entered the nucleus to induce the expression of related anti-apoptotic genes, and promoted the progression of cancer. **(B)**. In pancreatic tissue, OTUB2 cleaved the K63 polyubiquitin chain of NEMO guided by TRAF6, and the K63 polyubiquitin chain of NEMO was a sufficient condition for the activation of the IKK complex. Therefore, the NF-kappaB pathway was blocked in the IKK complex. Different from cancer tissue, physiologically, the expression of target genes induced by NF-kappaB tended to cause the apoptosis of pancreatic *β*-cells, so OTUB2 protected the only pancreatic *β*-cells that secreted hypoglycemic hormones in the human body.

The nuclear factor kappa B (NF-kappaB) pathway is widely considered to play a positive role in cancers, which can up-regulate the expression of anti-apoptotic genes to combat the adverse reactions caused by inflammation (Hoesel and Schmid, 2013). Inhibitor of kappaB (IkappaB) kinase (IKK, composed of IKKα, IKKβ, and NEMO) is an important signal molecule complex in the NF-kappaB pathway, which is activated by the phosphorylated modification of upstream molecule transforming growth factor beta-activated kinase 1 (TAK1) (Wang et al., 2001; Chen et al., 2006). TNF receptor-associated factor 6 (TRAF6) has dual effects on the IKK complex. On the one hand, TRAF6 can modify the Lysine-63 polyubiquitin chain on TAK1-binding protein 2 (TAB2) to recruit TAK1 for signal transduction; on the other hand, it can directly polyubiquitinate the NF-kappaB essential modulator (NEMO) with the Lysine-63 linked type (Singhirunnusorn et al., 2005; Chen et al., 2006). Once the IKK complex is activated, IkappaBα is phosphorylated, followed by proteasomal degradation of IkappaBα mediated by canonical the K48 polyubiquitin chain (Hoesel and Schmid, 2013). At this point, the binding of IkappaBα to NF-kappaB is greatly weakened or even disappeared. These processes greatly inhibit the cytoplasmic localization of NF-kappaB protein by IkappaBα, making NF-kappaB transcription factors (p65 + p50) enter the nucleus to activate anti-apoptotic genes expression (Figure 3A).

Based on the above functions of TRAF6 in the NF-kappaB pathway, Ma et al. proposed that OTUB2 in thyroid cancer could guide the deubiquitination of TRAF6 and promote the activation of the NF-kappaB pathway, which seemed to be consistent with previous work (Li et al., 2010; Ma and Sun, 2019). Moreover, the expression level of OTUB2 showed an antagonistic trend with the amount of miR-29a-3p, that is, miR-29a-3p could inhibit the expression of OTUB2 (Ma and Sun, 2019). It can be found that ubiquitination can guide a variety of physiological functions. The K63 polyubiquitin chain driven by TRAF6 is not a traditional classical proteasome inhibition pathway symbol, and it seems that the effect of OTUB2 on TRAF6 is to avoid the K48-mediated degradation of TRAF6 according to the experimental results. TRAF6 in the NF-kappaB pathway is closely related to OTUB2, and its core molecule phosphorylated p65 protein (one of the proteins constituting NF-kappaB dimer) also has a specific correlation with OTUB2. In liver cancer, the knockout of OTUB2 suppressed the phosphorylated p65 level in liver cancer cells (Gu et al., 2020). This result had also been confirmed in papillary thyroid cancer: the overexpression of OTUB2 can restore the level of phosphorylated p65 (Ma and Sun, 2019). Unfortunately, the exact molecular mechanism by which OTUB2 reduces the content of phosphorylated p65 remains unknown. It is a consensus that the phosphorylation of p65 is a necessary condition for nuclear localization of NF-kappaB to play a cancer-promoting effect (Viatour et al., 2005). Therefore, OTUB2 may be involved in the K48-mediated degradation of proteins that are responsible for the phosphorylation of p65, such as casein kinase 2 (CK2) (Bird et al., 1997; Wang et al., 2000), eventually, OTUB2 perhaps indirectly lead to the reduction of phosphorylation modification of p65. Of course, this approach is worth exploring with experiments.

#### 3.1.3 The Akt/mTOR Pathway

The protein kinase B/mammalian target of rapamycin (Akt/mTOR) pathway is necessary to regulate cell proliferation and invasion, and its pathological abnormal activation is inseparable from tumor invasion and metastasis (Laplante and Sabatini, 2012). There is evidence that OTUB2’s veil in the Akt/mTOR pathway had been partially lifted (Li et al., 2019a). The study first observed that in non-small cell lung cancer, the overexpression of OTUB2 protein promoted the Warburg effect (enhanced cellular glycolysis). Then the interaction between OTUB2 and U2 small nuclear RNA auxiliary factor 2 (U2AF2) was found through the immunoprecipitation method. Finally, in the case of U2AF2 gene knockout, the content of p-Akt and p-mTOR dropped sharply, confirming that OTUB2 stabilized the activation of the Akt/mTOR pathway by deubiquitinating U2AF2 (Li et al., 2019a). Apparently, the type of deubiquitination mediated by OTUB2 is the K48 polyubiquitin chain, which aims to avoid the degradation of U2AF2 by the proteasome to preserve the content of U2AF2. OTUB2 is linked to the Akt/mTOR pathway by targeting U2AF2, unfortunately, the molecular mechanism of U2AF2 in non-small cell lung cancer is still unclear. But one research demonstrated that U2AF2 had a sign of elevation in non-small cell lung cancer (Zhang et al., 2016).

Intriguingly, a downstream target of Akt is IKKα, and the phosphorylation of IKKα can trigger the NF-kappaB pathway (Viatour et al., 2005). As mentioned above, OTUB2 can stably activate the NF-kappaB pathway through K48 type deubiquitination of TRAF6 and maintenance of phosphorylated p65, resulting in pathological manifestations of cancer. It seems that the Akt pathway can also trigger other NF-kappaB pathways. Therefore, OTUB2 can present a diversified way of regulating related signal pathways by acting on different proteins, depending on the crosstalk linkage between cell signal transduction pathways.

### 3.2 The Duality of OTUB2 in Normal Development and Physiological Processes by Influencing Cell Signaling Pathways

#### 3.2.1 The Hedgehog Pathway

**FIGURE 4:**
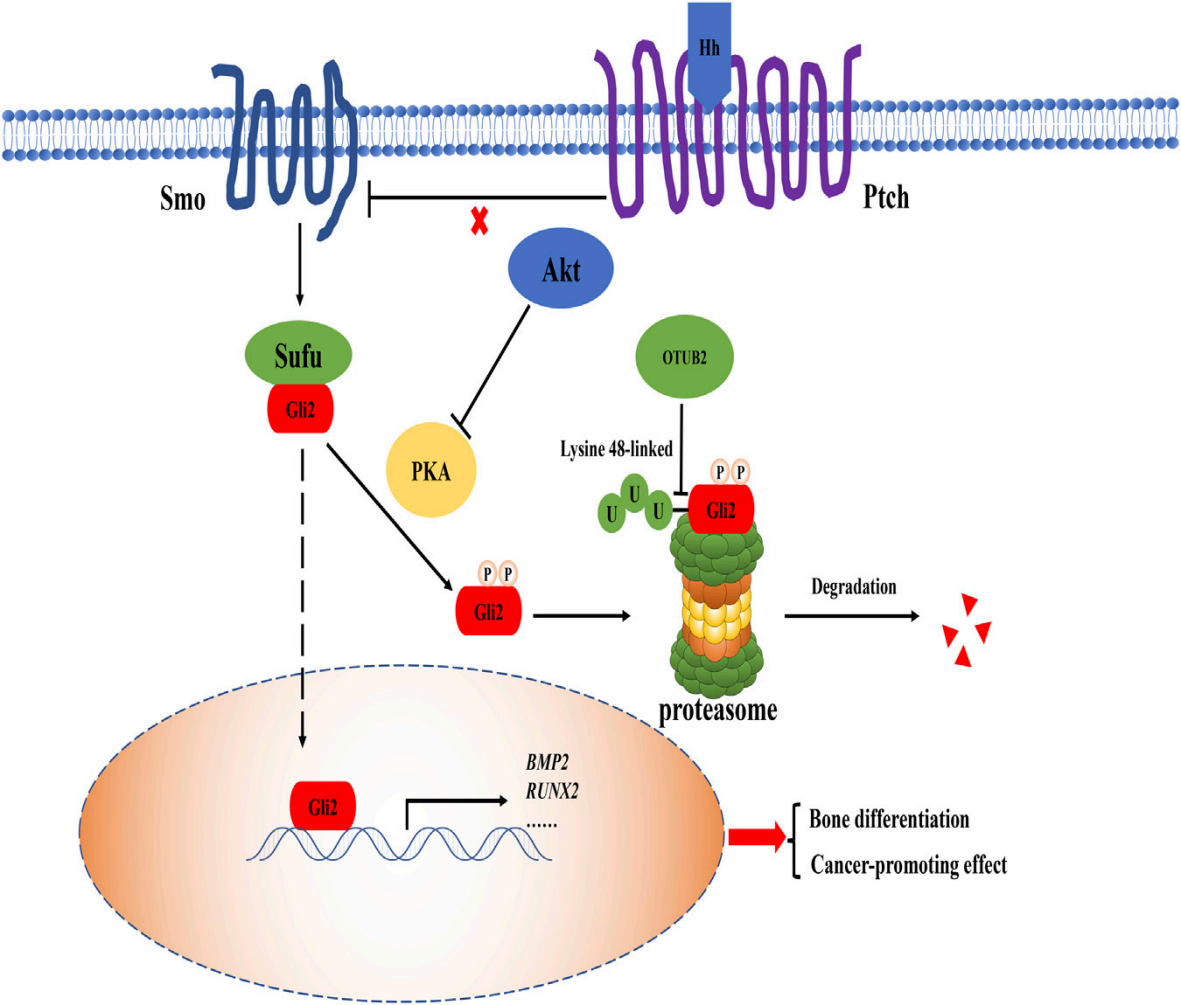
The role of OTUB2 in the Hh pathway. When Hh protein bound to the patched (Ptch) receptor, the original inhibition of Ptch on the smoothened (Smo) co-receptor was released, and the Hh pathway was activated. Suppressor of fused (Sufu) protein separated from Gli2, thus Gli2 entered the nucleus as a transcription factor to maintain the expression of related bone differentiation genes such as BMP2, Runt-related transcription factor 2 (RUNX2). The overactivation of the Hh signaling pathway resulted from direct deubiquitination of Gli2 by OTUB2 (i.e. cleavage of the K48 polyubiquitin chain as a degradation signal marker) and was closely related to the indirect blockage of Gli2 degradation by Akt. Therefore, OTUB2 may promote the occurrence of cancer effect.

The Hedgehog (Hh) signaling pathway plays a dominant role in regulating the proliferation, differentiation, and maintenance of growth plate chondrocytes in the bone growth period (Haraguchi et al., 2019). This pathway functions by regulating genes expression through the nuclear localization of the transcription factor Gli family (Rimkus et al., 2016). Bone morphogenetic proteins (BMP) superfamily is one of the expression products of specific genes that induce osteogenic differentiation, and it can achieve this goal by activating the Smad pathway (Chen et al., 2012; Rui et al., 2012). It was reported that glioma-associated oncogene protein-2 (Gli2) is at risk of degradation in the cytoplasm (Li et al., 2018). The report had revealed that knocking out OTUB2 can reduce the level of Gli2, and the treatment with the proteasome inhibitor MG-132 can restore Gli2 expression. Meanwhile, the interaction between OTUB2 and Gli2 was determined by immunoprecipitation (Li et al., 2018). Under the action of Hh and Smo (upstream molecules of the Hh pathway) agonists, the lack of OTUB2 inhibited the expression of bone differentiation marker BMP2, which proved that OTUB2 was indeed used as a degradation inhibitor to rescue the reduction of Gli2 and indirectly activate the Hh pathway (Figure 4) (Li et al., 2018). Like OTUB2’s deubiquitination-type function in many other pathways, OTUB2 cleaves the K48 polyubiquitin chain to ensure the presence of Gli2. As for the downstream effects of the Hh pathway, since the Hh pathway is often multiple and complex, it is not completely dependent on OTUB2. It can just be said that OTUB2 provides new insight into the regulation of the Hh pathway.

There is no doubt that OTUB2 plays a positive role in the Hh pathway to promote osteogenic differentiation and maintain normal human development. However, the over-activated Hh pathway is an ominous marker of cancer progression. Several core factors of the Hh pathway, including Gli2, are highly expressed in cancer (Roberts et al., 1989). Recently, there was another view that the Hh pathway and Akt/mTOR pathway could affect each other. On the one hand, the increase of mTOR activity led to the augment of Gli2 transcription factor in the Hh signaling pathway; on the other hand, the Hh signaling pathway positively regulated mTOR complex 1 (mTORC1) activity by up-regulating the homeodomain protein NK2 homeobox 2 (NKX2.2), creating a vicious circle between the two pathways to accelerate cancer progression (Klein et al., 2019; Larsen and Møller, 2020). There was also evidence that Akt can regulate protein kinase A (PKA) so that Gli2 was prevented from being phosphorylated and further degraded by the K48 polyubiquitin chain (Riobó et al., 2006). OTUB2 acts as an activator by deubiquitinating related proteins with the K48 polyubiquitin chain in both pathways. From this perspective, OTUB2 is not conducive to the normal physiological function of cells.

#### 3.2.2 The NF-kappaB Pathway

The NF-kappaB pathway has previously been described in cancers as it could induce the expression of related anti-apoptotic genes to antagonize the apoptosis caused by inflammation. Conversely, the NF-kappaB pathway has the effect of promoting apoptosis in pancreatic *β*-cells, thus showing its particularity (Rink et al., 2012). This mainly depends on the stimulation of the environmental factors in which the cells are located (Ortis et al., 2012). More than this special feature, if OTUB2 interacts with TRAF6, the result should benefit the NF-kappaB pathway. However, a study unexpectedly found that under the interference of siRNA on OTUB2, the activity of the NF-kappaB pathway of pancreatic *β*-cells was enhanced, and the amount of insulin secretion was inhibited, indicating that a large number of pancreatic *β*-cells were apoptotic (Beck et al., 2013). It is inconsistent with other studies on OTUB2’s function of promoting NF-kappaB pathway activity.

What is quite explored is that OTUB2 may antagonize the function of TRAF6 which acts as an E3 ubiquitin ligase in pancreatic *β*-cells (Beck et al., 2013). It is well known that TRAF6 can modify NEMO together with ubiquitin-conjugating enzyme 13/ubiquitin E2 variant 1a (Ubc13/Uev1A) and catalyze the transfer of the unique polyubiquitin chain linked by K63, contributing a lot to the activation of the IKK complex (Deng et al., 2000). NEMO’s K63 ubiquitination is unique because of its novel regulatory function (second messengers), unlike traditional ubiquitination (degradation function) (Deng et al., 2000). We can assume that if OTUB2 deubiquitinated NEMO, the IKK complex would not work, and the NF-kappaB pathway would collapse. It is most likely that OTUB2 relies on cell specificity and abnormally inhibits the activity of the NF-kappaB pathway by deubiquitinating NEMO with the K63 polyubiquitin chain (Figure 3B).

Surprisingly, in the NF-kappaB pathway, NEMO can be activated by the K63 polyubiquitin chain modification. In recent years, linear ubiquitin chains have also been shown to be able to modify NEMO by linear ubiquitin chain assembly complex (LUBAC) in response to the stimulation of tumor necrosis factor and interleukin-1 (Heger et al., 2018). However, one study is skeptical about the de-linear ubiquitination function of OTUB2 to NEMO, and the types of deubiquitination of OTUB2 are limited to the K48, the K63, and the K11 polyubiquitin chain (Mevissen et al., 2013). Furthermore, the generation and cleavage of the linear ubiquitin chain seem to be uniquely selective. At present, only the LUBAC is involved in the formation of the linear ubiquitin chain of substrate proteins, and only the cleavage of linear ubiquitin chains has been discovered in OTULIN (Mevissen et al., 2013; Aksentijevich and Zhou, 2017). Therefore, the regulation of NEMO ubiquitination and its deubiquitination varies in different cells and their microenvironments.

### 4 OTUB2’s Effect on Physiology

#### 4.1 The Relationship Between OTUB2 and DNA Damage Repair

DNA double-strand break (DSB) is the most harmful type of DNA damage and occurs almost all the time in cells (Li et al., 2019b). Homologous recombination (HR) ensured high fidelity repair after DNA double-strand damage (Wright et al., 2018). Although the non-homologous end joining (NHEJ) pathway can lead to slight changes in the DNA sequence at the cleavage site, it can preserve the stability of the genome early and rapidly (Hefferin and Tomkinson, 2005). The appropriate choice between HR and NHEJ is crucial in different contexts, and OTUB2 provides us with a new perspective (Kato et al., 2014; Ceccaldi et al., 2016). OTUB2 can specifically cleave the RNF8-mediated the K63 ubiquitin chain of lethal (3) malignant brain tumor-like protein 1 (L3MBTL1) (Kato et al., 2014). The K63 ubiquitination of L3MBTL1 is the prerequisite for the accumulation of p53 binding protein 1 (53BP1) and the protection of the DSB broken end. This inhibits DNA end resection, that is, HR repair is not applicable in this case, thus providing conditions for NHEJ repair (Acs et al., 2011). The deletion of OTUB2 leads to the bias of DNA double-strand repair selection to NHEJ. Here, the K63 ubiquitination is a DNA repair regulated signal. OTUB2 directly antagonizes RNF8-mediated K63-linked ubiquitin chains and thus participates in DNA repair regulation. The effect is direct, rather than monotonically avoiding protein degradation like the deubiquitination function of OTUB2 in the cell signaling pathway. It is the specificity of the K63 ubiquitin chain and the properties of the substrate that endow OTUB2 with an indelible role in DNA damage repair.

Similarly, overexpression of OTUB2 in endometrial carcinoma can indirectly increase Rad51 expression by interacting with YAP/TAZ to promote HR (Wan et al., 2020). However, this HR in endometrial cancer tissues seems to be pathological (Wan et al., 2020). Cancer cells rely on HR to maintain their genetic stability and avoid apoptosis, which generally aggravates cancer progression.

### 4.2 The Relationship Between OTUB2 and Immunity

The strong link between OTUB2 and immunity was reported by Li et al. that OTUB2 could deubiquitinate TRAF6 to inhibit viral-induced type I interferons (IFN-1) activation (Li et al., 2010). IFN-1 are beneficial to cell antiviral reaction and acquired immunity such as cell differentiation into specific immune cells (Schreiber, 2017). Therefore, OTUB2 is involved in the physiological process of immunosuppression. In addition, as the mechanism of various cell signaling pathways in the immune response is gradually revealed, OTUB2 can perform in the immune response to some extent by regulating cell signaling pathways. For example, OTUB2 can up-regulate phosphorylated p65 levels, thereby activating the NF-kappaB pathway (Gu et al., 2020). In the inflammatory response, the NF-kappaB pathway enables the expression of adhesion molecules on the cell surface, mediates the mutual recognition of leukocytes and vascular endothelial cells in the circulatory system, and directs the chemotaxis of leukocytes to the infected area (Eck et al., 1993; Alcamo et al., 2001). In acquired immunity, T cells and B cells often depend on the anti-apoptotic proteins B-cell lymphoma-2 (BCL-2) and B-cell lymphoma-extra large (BCL-XL) mediated by NF-kappaB transcription factors to maintain their development (Sentman et al., 1991; Feng et al., 2004).

In particular, in the Hippo pathway of tumor cells, programmed cell death ligand 1 (PD-L1) induced by YAP/TAZ is a cell membrane surface marker on tumor cells, which usually binds to homologous receptors on cytotoxic T lymphocytes (CTL), induces T cell depletion and prevents CTL-mediated tumor-specific killing. It mediates tumor immunosuppression and is the culprit that makes tumor cells escape from the body’s immunity without being cleared by immune cells. (Nguyen and Yi, 2019). PD-L1-induced tumor immunosuppression has been implicated in OTUB1 (Zhu D. et al., 2021). As a homolog of OTUB1, OTUB2 can also help YAP/TAZ enter the nucleus to trigger downstream genes expression. However, the cancer-promoting effect of OTUB2 has been widely known, there is no report on the relationship between OTUB2 and tumor immunity. Whether OTUB2 protects tumor cells through immunosuppression and thus deteriorates tumor progression will be a new research direction.

## 5 Discussion

In this review, we briefly mentioned the molecular structure of OTUB2 and its function of ubiquitin cleavage and systematically expounded the physiological control of OTUB2 on cell development and the pathological acceleration of tumors or other diseases by OTUB2 from the perspective of cell signaling pathways (Table 1). Moreover, we also clarified the physiological function of OTUB2 from the standpoint of DNA damage repair and immunology, thus supplementing some conjecture of OTUB2 carcinogenesis and opening a new way for future research direction. In general, OTUB2 mediates the deubiquitination of substrate proteins, and the targets of substrate proteins can be diversified, so OTUB2 has its place in a variety of cytological functions. For example, in gastric cancer cells, OTUB2 can deubiquitinate the lysine specific demethylase 1A (KDM1A, not only can demethylate histones, but also p53, DNA methyltransferase, etc.) to promote gastric cancer cell proliferation, migration, and invasion (Huang et al., 2007; Lan et al., 2008; Wang et al., 2009; Liu et al., 2021). However, due to insufficient research and the molecular mechanism of some diseases has not yet been elucidated, the current understanding of OTUB2 is not comprehensive. For instance, the integration of gene tissue microarrays suggests that OTUB2 is a specifically expressed motor neuron degeneration signal factor in amyotrophic lateral sclerosis (Kudo et al., 2010). Nevertheless, the research is restricted to a qualitative analysis.

**TABLE 1:** Target protein deubiquitination mediated by OTUB2.

Target substrate	Polyubiquitin chain type	Result of ubiquitination	Affected pathway or event	Effect	References
YAP/TAZ	K48	Degradation	Hippo	Enhance cancer cell metastasis	Zhang et al. (2019)
TRAF6	K48	Degradation	NF-kappaB	Promote cancer cell proliferation, growth, and invasion	Ma and Sun, (2019)
U2AF2	K48	Degradation	Akt/mTOR	Promote Warburg effect and cancer occurrence	Li et al. (2019a)
Gli2	Unknown	Degradation	Hedgehog	Promote osteogenic differentiation but worsen cancer progression	Li et al., (2018), Roberts et al. (1989)
NEMO	K63	Signal transmission	NF-kappaB	Inhibit pancreatic *β*-cells apoptosis	Beck et al. (2013)
L3MBTL1	K63	Selection of DNA damage repair pathways	DNA damage repair	Promote HR repair	Kato et al. (2014)

What’s more, the function of OTUB2 is well known for its deubiquitination, and the deubiquitination is relatively diverse in terms of the polyubiquitin chain. This suggests that the substrate proteins targeted by OTUB2 seem to be more extensive than that of OTUB1 with specific cleavage of the K48 polyubiquitin chain. In addition, according to the existing knowledge, our understanding of the deubiquitination function of OTUB2 is limited to the deubiquitination types of the K48 and the K63. However, it should be emphasized that OTUB2 has the cleavage effect of the K11 polyubiquitin chain, which may also make OTUB2 play an unusual role in controlling the cell mitosis cycle (Wickliffe et al., 2011; Mevissen et al., 2013). Future research on the K11 cleavage function of OTUB2 may deeply understand OTUB2 to view the relationship between OTUB2 and disease from a new perspective.

So far, most of the reports about OTUB2 in human diseases are adverse, and only a few indicated that the knockout of OTUB2 can lead to osteogenic differentiation disorders and pancreatic *β*-cell apoptosis. The basal level of OTUB2 expression in normal tissue is self-evidently important for development and physiological functions, but relatively speaking, targeting excessive OTUB2 in diseases such as cancers may be a promising therapeutic target. The inhibition of OTUB2 expression can be carried out in the following two directions. One is precisely targeting upstream molecules that can regulate the expression of OTUB2, such as miR-29a-3p in thyroid cancer (Ma and Sun, 2019). The biggest challenge is that systematic research on how to control the expression of OTUB2 is not comprehensive, and miRNAs are often tissue-specific (Mishra et al., 2016). The other is the inhibitor that directly inhibits the catalytic activity of OTUB2, such as a ligand probe that can covalently bind to Cys residue of the catalytically active triad of OTUB2, and its selectivity is much higher than other DUBs (Resnick et al., 2019). Although OTUB2 is theoretically overexpressed in diseases such as cancers, its basic expression in normal cells should also be considered. However, the expression of OTUB2 is rather low in normal tissue, and targeted inhibition will not affect cell apoptosis, differentiation at the cellular level (Liu et al., 2021). This may be due to the powerful compensatory supplementation of DUBs *in vivo*. Therefore, OTUB2 is a functional cysteine protease molecule that can be studied and targeted for therapy.

More importantly, the exact molecular mechanism of OTUB2 in disease is unknown. Although OTUB2 is similar to other DUBs to some extent, the molecular structure of OTUB2 is still different from that of normal DUBs (Nanao et al., 2004). In the future, the target proteins of OTUB2 can be further identified by analogy with other substrates acted on by DUBS. It is also a feasible method to detect the specific substrate of OTUB2 by bioinformatics analysis. In this way, the function of OTUB2 *in vivo* and the mechanism of OTUB2 in diseases can be viewed at a deeper level.

In general, the impact of OTUB2 on the body needs to be critically viewed. First of all, it is irrefutable for the functional properties of protein deubiquitination of OTUB2, but protein ubiquitination can be multifunctional, such as K48 ubiquitination-guided degradation (Grice and Nathan, 2016), K63-guided messenger transmission (Cohen, 2018), and K11- guided regulation of cell division (Wickliffe et al., 2011). In addition, OTUB2 has a wide range of cleavage properties for polyubiquitin chains so that OTUB2 can mediate different physiological functions. Secondly, OTUB2 greatly regulates proteins related to cell transduction pathways. Besides, cell signaling transduction pathways are always complex and crosstalk. Last but not least, the tissue cell specificity of OTUB2 is also intriguing. OTUB2 determines cell differentiation in osteoblasts, inhibits *β*-cell death in pancreatic islet tissue, and accelerates cancer progression in some cancer tissues. OTUB2 has both positive and negative effects on the human body. Therefore, a comprehensive view of the role of OTUB2 in cells must be related to the environment and physiological state of cells.

## 6 Conclusion

OTUB2 acts on different substrates and participates in a variety of cell signaling pathways or cell physiological activities to control the progression of the diseases. The deubiquitination types of OTUB2 (at least in the K11 polyubiquitin chain) have not been fully studied. Therefore, other unknown substrate proteins and detailed deubiquitination type regulation mechanism of OTUB2 are hot topics worthy of further study, and OTUB2 may become a therapeutic target for some diseases.
